# Liniment for Bruises

**Published:** 1891-10

**Authors:** 


### Liniment for Bruises.

R. Tr. capsicum.........................................2 parts.
T. myrrh.................................................2 parts.
Tr. opium...............................................2 parts.
Tr. guaiac..............................................1 part.
Sps. champhor.......................................8 parts.

M. This is similar to Perry Davis' Pain Killer.

A much more powerful one is:

R. Tr. aconite.........................................
Tr. opium...............................................
Chloroform aa........................................1 part.

M. Shake well before using.—H. M. Whelpley, in *Cincinnati Medical Journal.*

